# Effects of high EPA and high DHA fish oils on changes in signaling associated with protein metabolism induced by hindlimb suspension in rats

**DOI:** 10.14814/phy2.12958

**Published:** 2016-09-20

**Authors:** Gabriel Nasri Marzuca‐Nassr, Kaio Fernando Vitzel, Luís Gustavo De Sousa, Gilson M. Murata, Amanda Rabello Crisma, Carlos Flores Rodrigues Junior, Phablo Abreu, Rosângela Pavan Torres, Jorge Mancini‐Filho, Sandro M. Hirabara, Philip Newsholme, Rui Curi

**Affiliations:** ^1^ Department of Physiology and BiophysicsInstitute of Biomedical SciencesUniversity of São PauloSão PauloBrazil; ^2^ Massey Institute of Food Science and TechnologyCollege of HealthMassey UniversityAlbanyNew Zealand; ^3^ Department of Lipids Laboratory, Food Science & NutritionFaculty of Pharmaceutical ScienceUniversity of São PauloSão PauloBrazil; ^4^ Institute of Physical Activity Sciences and SportCruzeiro do Sul UniversitySão PauloBrazil; ^5^ School of Biomedical SciencesCHIRI BiosciencesCurtin UniversityPerthAustralia

**Keywords:** Hindlimb suspension, muscle atrophy, omega‐3 fatty acids, protein synthesis/degradation

## Abstract

The effects of either eicosapentaenoic (EPA)‐ or docosahexaenoic (DHA)‐rich fish oils on hindlimb suspension (HS)‐induced muscle disuse atrophy were compared. Daily oral supplementations (0.3 mL/100 g b.w.) with mineral oil (MO) or high EPA or high DHA fish oils were performed in adult rats. After 2 weeks, the animals were subjected to HS for further 2 weeks. The treatments were maintained alongside HS. At the end of 4 weeks, we evaluated: body weight gain, muscle mass and fat depots, composition of fatty acids, cross‐sectional areas (CSA) of the soleus muscle and soleus muscle fibers, activities of cathepsin L and 26S proteasome, and content of carbonylated proteins in the soleus muscle. Signaling pathway activities associated with protein synthesis (Akt, p70S6K, S6, 4EBP1, and GSK3‐beta) and protein degradation (atrogin‐1/MAFbx, and MuRF1) were evaluated. HS decreased muscle mass, CSA of soleus muscle and soleus muscle fibers, and altered signaling associated with protein synthesis (decreased) and protein degradation (increased). The treatment with either fish oil decreased the ratio of omega‐6/omega‐3 fatty acids and changed protein synthesis‐associated signaling. EPA‐rich fish oil attenuated the changes induced by HS on 26S proteasome activity, CSA of soleus muscle fibers, and levels of p‐Akt, total p70S6K, p‐p70S6K/total p70S6K, p‐4EBP1, p‐GSK3‐beta, p‐ERK2, and total ERK 1/2 proteins. DHA‐rich fish oil attenuated the changes induced by HS on p‐4EBP1 and total ERK1 levels. The effects of EPA‐rich fish oil on protein synthesis signaling were more pronounced. Both EPA‐ and DHA‐rich fish oils did not impact skeletal muscle mass loss induced by non‐inflammatory HS.

## Introduction

Skeletal muscle mass loss occurs in response to disuse (e.g., immobilization, denervation or lack of mechanical load), aging (sarcopenia), starvation, and severe catabolic states (such as cancer cachexia and sepsis). Muscle atrophy is associated with a decrease in activity of protein synthesis and/or an increase in protein degradation signaling. These two pathways are highly regulated via growth factor effects and are interrelated (Jackman and Kandarian 2004). Several strategies have been investigated to treat skeletal muscle atrophy, including electrical stimulation (Boonyarom et al. 2009; Dirks et al. 2015), physical exercise (Fujino et al. 2009; Morimoto et al. 2013; Liu et al. 2014), and dietary supplementation (e.g., leucine, isoleucine, valine, creatine, and L‐carnitine) (Magne et al. 2013; Wall and van Loon 2013; de Campos‐Ferraz et al. 2014; D'Antona et al. 2014). Fish oil has been postulated, in review articles, as a potential attenuating agent of skeletal muscle atrophy (Magne et al. 2013; Wall and van Loon 2013; D'Antona et al. 2014). In this study, we investigated the effects of either eicosapentaenoic (EPA)‐rich or docosahexaenoic (DHA)‐rich fish oil on signaling pathways associated with protein synthesis and degradation in an experimental condition of intense skeletal muscle mass loss.

Fish oil is rich in two omega‐3 fatty acids, EPA and DHA. Fish oils have been reported to reduce severity of diseases such as diabetes (Yanai et al. 2015), AIDS (Paranandi et al. 2014), cancer cachexia (Tisdale 2007), chronic heart failure (Holdsworth et al. 2014), chronic lung disease (Miyata and Arita 2015), and sepsis (Gultekin et al. 2014). These diseased conditions are associated with marked loss of skeletal muscle mass. In vivo treatment with omega‐3 fatty acids increases activity of signaling pathways associated with protein synthesis (Protein kinase B [Akt], mammalian target of rapamycin [mTOR], p70 ribosomal protein S6 kinase [p70S6K]) (Gingras et al. 2007; You et al. 2010a,b; Smith et al. 2011a,b; Kamolrat et al. 2013) and reduces protein degradation (atrogin‐1/muscle atrophy F‐box protein [MAFbx], and muscle RING finger 1 [MuRF1]) (You et al. 2010b). However, there are relatively few studies on signaling pathways associated with protein synthesis and degradation in conditions of global skeletal muscle mass loss. A cod liver oil‐rich diet attenuated soleus muscle atrophy in a limb immobilization animal model (You et al. 2010b). Supplementation with EPA by gavage also attenuated soleus muscle atrophy in cancer cachexia (Whitehouse et al. 2001). The experimental protocol used to induce skeletal muscle mass loss in these studies was designed to model chronic disuse conditions. Comparative effects of DHA‐ and EPA‐rich fish oils on intracellular signaling have not been previously investigated.

This study was undertaken to determine the effects of treatment with high EPA or high DHA fish oils on skeletal muscle signaling pathways associated with protein synthesis and degradation in rats submitted to hindlimb suspension (HS)‐induced atrophy. We examined and compared the effects of EPA‐ and DHA‐rich fish oils on signaling pathways associated with protein synthesis and degradation in a condition of intense skeletal muscle mass loss induced by hindlimb suspension, a model of skeletal muscle disuse as occurs in bed rest or spaceflight conditions in humans.

## Materials and Methods

### Animals

Eight‐week‐old male Wistar rats were obtained from the Animal Facility of the Department of Physiology and Biophysics, Institute of Biomedical Sciences, University of São Paulo. The animals were maintained under standard conditions: light/dark cycle 12 h each and food and water ad libitum (daily consumption was recorded). All experimental procedures were performed in accordance with the Guide for Care and Use of Laboratory Animals (Institute of Laboratory Animal Resources, National Academy of Sciences, Washington, DC). Ethics Committee of the Institute of Biomedical Sciences, University of São Paulo, approved this study.

### Experimental study design

During the first 3 days of the experimental period, the animals were left to adapt to individual cages. Afterwards, rats were divided into the following groups: mineral oil supplemented (MO‐C, *n* = 12); mineral oil supplemented and hindlimb suspension (MO‐HS, *n* = 12); high EPA fish oil supplemented (EPA‐C, *n* = 12); high EPA fish oil supplemented and hindlimb suspension (EPA‐HS, *n* = 12); high DHA fish oil supplemented (DHA‐C, *n* = 12) and high DHA fish oil supplemented and hindlimb suspension (DHA‐HS, *n* = 12). Rats received a daily oral supplementation (by gavage) of mineral oil (MO) or high EPA or high DHA fish oils, 0.3 mL/100 g b.w., during 4 weeks. The high EPA (68% EPA and 16% DHA; EPA/DHA ratio 4.3:1) and high DHA (61% DHA and 11% EPA; EPA/DHA ratio 1:5.7) fish oils were obtained from Naturalis^®^, São Paulo, Brazil. The fatty acid composition of the fish oils is in Table 1. Considering a fish oil density of 0.92 g/cm^3^, the doses in grams were: 1.88 mg EPA and 0.44 mg DHA per g b.w. in the high EPA fish oil and 0.30 mg EPA and 1.68 mg DHA per g b.w. in the DHA‐rich fish oil. The animals were submitted to HS from the end of the second week of treatment on. Rats were then maintained in HS and supplementations with fish oils or mineral oil concomitantly for 2 weeks to complete the full period of 4‐week experimentation. After 4 weeks, animals were anesthetized using ketamine (90 mg/kg b.w.) and xylazine (10 mg/kg b.w.) by intraperitoneal administration. Animals were then weighed and killed by exsanguination. Soleus muscles of both limbs were removed and stored at −80°C for histological and molecular analysis. Gastrocnemius, tibialis anterior, and extensor digitorum longus (EDL) muscles, as well as subcutaneous, epididymal, retroperitoneal, and mesenteric adipose tissue depots were removed and weighed.

**Table 1:** Fatty acid composition (% of the total fatty acids) of the high EPA and high DHA fish oils. Values are presented as mean ± SD of three determinations

Fatty acid	Name	High EPA fish oil	High DHA fish oil
14: 0	Myristic	—	—
16: 0	Palmitic	—	1.603 ± 0.097
16: 1 (n‐7)	Hexadecenoic	0.558 ± 0.034	0.437 ± 0.379
17: 0	Margaric	—	—
17: 1 (n‐7)	Heptadecenoic	—	—
18: 0	Stearic	—	1.613 ± 0.037
18: 1 (n‐9)	Oleic	0.887 ± 0.008	4.779 ± 0.006
18: 1 (n‐7)	Vaccenic	—	1.189 ± 0.029
18: 2 (n‐6)	Linoleic	0.527 ± 0.047	1.707 ± 0.039
20: 0	Eicosanoic	—	—
20: 1 (n‐9)	Eicosenoic	—	0.807 ± 0.005
18: 3 (n‐6)	*γ*‐Linolenic	—	—
18: 3 (n‐3)	*α*‐Linolenic	0.040 ± 0.034	—
22: 0	Docosanoic	0.025 ± 0.0024	—
20: 2	Eicosadienoic	2.848 ± 0.053	0.800 ± 0.017
20: 3 (n‐6)	Eicosatrienoic	—	—
20: 4 (n‐6)	Arachidonic	2.678 ± 0.043	2.474 ± 0.015
20: 5 (n‐3)	Eicosapentaenoic	67.695 ± 0.125	10.705 ± 0.045
22: 2 (n‐6)	Docosadienoic	1.007 ± 0.004	0.780 ± 0.025
24: 1 (n‐9)	Nervonic	—	0.586 ± 0.008
22: 5 (n‐6)	Docosapentaenoic	0.702 ± 0.026	4.270 ± 0.105
22: 5 (n‐3)	Docosapentaenoic	3.657 ± 0.079	5.078 ± 0.339
22: 6 (n‐3)	Docosahexaenoic	15.793 ± 0.592	60.928 ± 0.310
Unidentified		3.647 ± 0.242	3.025 ± 0.421
Totals	Saturated	—	3.216 ± 0.128
	Monounsaturated	1.445 ± 0.037	7.797 ± 0.401
	Polyunsaturated	94.907 ± 0.279	85.961 ± 0.594
	Omega‐6	(3.908 ± 0.064)	(8.451 ± 0.078)
	Omega‐3	(87.145 ± 0.392)	(76.711 ± 0.572)
	Omega‐6/Omega‐3	0.045	0.11

EPA, eicosapentaenoic acid; DHA, docosahexaenoic acid.

### Hindlimb suspension

HS is a well‐established experimental model for induction of skeletal muscle mass loss (Morey‐Holton and Globus 2002). Special cages were designed for the hindlimb suspension protocol. The animals were maintained in individual cages with tail attached on the top of the cage, using a tape, for suspending the hind limbs (30° suspension between the floor and the body of the animal) as reported by others (Thomason et al. 1987; Morey‐Holton and Globus 2002). Suspended animals were free to walk using the front limbs and to obtain food and water ad libitum. This experimental model mimics muscle disuse that occurs in conditions of bed rest, hospitalization or spaceflight. In order to detect stress, rat‐tail clinical feature, hair, eyes, and facial appearances of the animals were evaluated twice a day. In any indication of pain or discomfort of the animals, the experiment was interrupted (Thomason et al. 1987; Morey‐Holton and Globus 2002). Daily water ingestion and daily food intake were measured once a week. Body weight variations were weekly recorded.

### Lipid extraction and determination of the composition of fatty acids in fish oils and gastrocnemius muscle by gas chromatography

The AOAC 996.06 (AOAC, 2005) and AOCS Ce 1j‐07 (AOCS, 2007) methods with the C13:0 fatty acid as standard in place of C11:0 were used. Fatty acid composition was determined in a GC 2010 plus equipped with automatic sample injector (AOC 20i), flame ionization detector, GC solution software (Shimadzu Co, Kyoto, Japan), and 100 m fused silica SP2560 capillary column 0.25 mm film (Supelco Park, Bellefonte, PA). Composition of fatty acids of high EPA and high DHA fish oils was expressed as percentage of total fatty acids from three determinations. The composition of fatty acids in the gastrocnemius muscle was expressed as g/100 g tissue wet weight (AOAC, 2005; AOCS, 2007).

### Histological analysis of the soleus muscle

Serial sections were performed in the central portion of the soleus muscles according to Bodine and Baar (2012). The slides were stained with hematoxylin and eosin (HE) for analysis of cross‐sectional area (CSA) of the whole soleus muscle and of the soleus muscle fibers (150 fibers per muscle). Photographs were taken using an optical microscope (Nikon Eclipse E1000, Fukuoka, Japan) attached to a digital camera (Nixon DXM 1200). The images were analyzed using the AxioVision program (version 4.8.1.0, Carl Zeiss Imaging Solutions, Jena, Germany).

### Cathepsin L activity measurement

Soleus muscle homogenization and evaluation of cathepsin L activity were performed using the Cathepsin L Activity Assay Kit (Abcam – ab65306, Abcam Inc., Cambridge, UK), according to the recommendations of the manufacturer and the method described by Jannig et al. (2014).

### 26S Proteasome activity determination

Soleus muscles were homogenized in a buffer containing 210 mmol/L D‐mannitol, 70 mmol/L sucrose, 5 mmol/L MOPS, and 1 mmol/L EDTA (pH 7.4). Centrifugation of the homogenate was performed for 15 min, at 12,000*g* and 4°C, and the supernatant used for determination of cytosolic proteins (Bradford 1976). The activity of the chymotrypsin site of the 26S portion of the proteasome was assessed by fluorometric assay, using the fluorogenic peptide Suc‐Leu‐Leu‐Val‐Tyr‐7‐amido‐4‐methylcoumarin (LLVY‐AMC, item #P802‐0005, Enzo Life Sciences, Farmingdale, NY), as described by Churchill et al. (2010) and Cunha et al. (2012). Measurements were performed in the absence and presence of epoxomicin (20 *μ*mol/L). The difference between the two rates was attributed to the proteasome activity (Cunha et al. 2012).

### Content of carbonylated proteins in the soleus muscle

The homogenates of the soleus muscles were prepared as described above with addition of 2% 2‐mercaptoethanol in the extraction buffer. The OxyBlotTM Protein Oxidation Detection Kit (S7150; Milipore, Billerica, MA) was used to assess the levels of carbonylated proteins according to the recommendations of the manufacturer. Amersham Imager 600 (Amersham/GE Healthcare, Uppsala, Sweden) was used for image acquisition and the bands quantitated using the Image J software (NIH, Bethesda, MD). Total loading of proteins for each sample, as indicated by the Ponceau S staining, was used to normalize results (Romero‐Calvo et al. 2010; Gilda and Gomes 2013; Fortes et al. 2016).

### Analysis of Akt, p70S6K, S6, 4EBP1, GSK3‐beta, atrogin‐1/MAFbx, MuRF1, and ERK 1/2 by western blot

Soleus muscles were homogenized in a buffer solution containing 10 mmol/L EDTA, 100 mmol/L Tris‐Base, 10 mmol/L sodium pyrophosphate, 100 mmol/L sodium fluoride, 10 mmol/L sodium orthovanadate, 2 mmol/L PMSF, and 0.01 mg/mL aprotinin. The samples were then sonicated for 30 sec at 4°C, treated with 1% Triton X‐100, and centrifuged at 20,000*g*, at 4°C, for 20 min. The total content of proteins of the supernatant of each sample was determined (Bradford 1976). Equal amounts of protein (40 *μ*g) from each sample were subjected to SDS‐PAGE according to Shapiro et al. (1967). The proteins were then transferred to a nitrocellulose membrane (Towbin et al. 1979) and had the nonspecific binding sites blocked by 1‐h incubation with 5% milk or 5% BSA solution.

**Figure 1:**
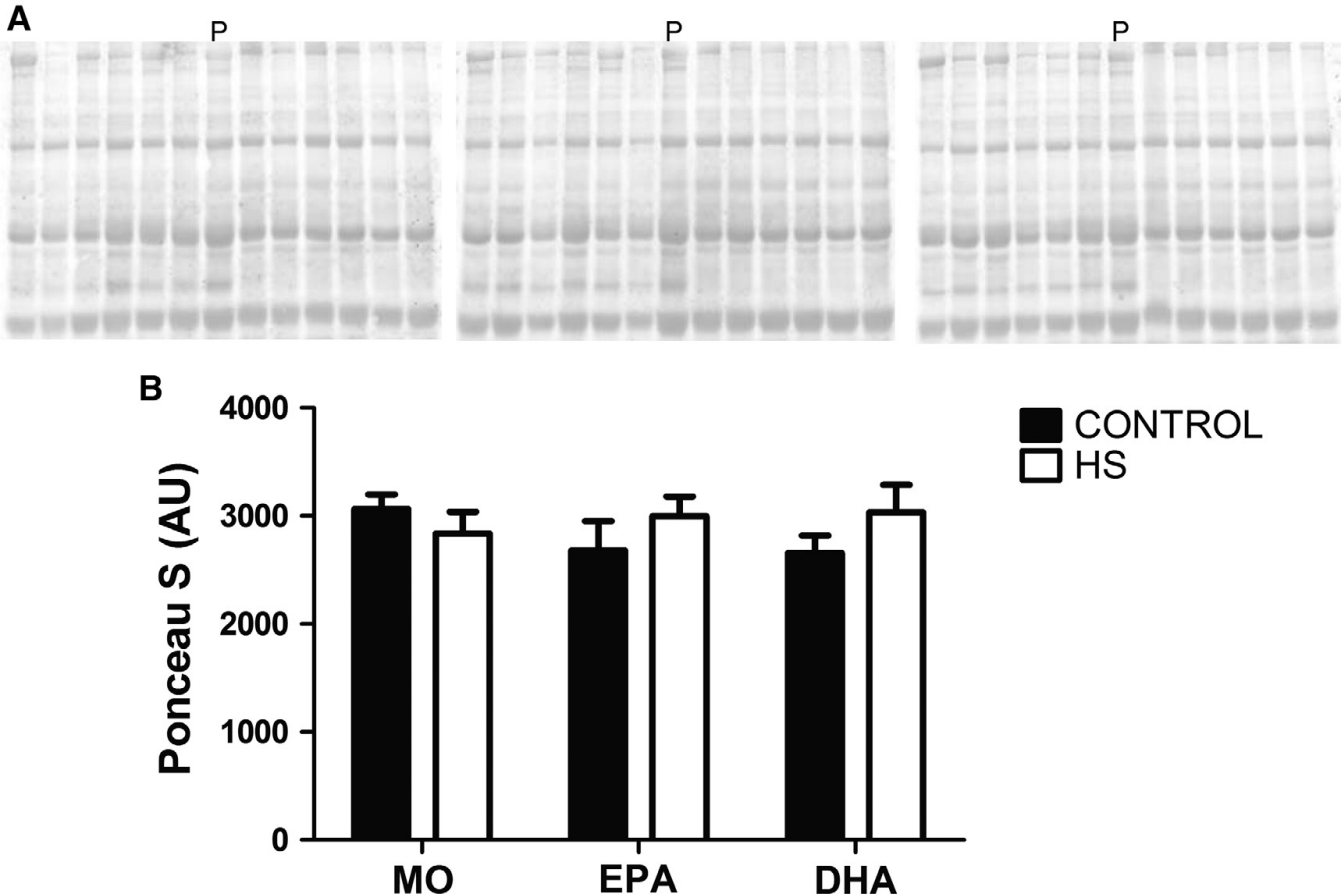
Quantitative analysis of western blot membranes stained with Ponceau S. (A) Images of the western blot membranes stained with Ponceau S used in this study. (B) Average quantitative analysis of Ponceau S staining. No significant differences were observed. The results were compared using two‐way ANOVA and Bonferroni post hoc test. MO, Mineral oil supplementation; EPA, High eicosapentaenoic acid fish oil supplementation; DHA, High docosahexaenoic acid fish oil supplementation; HS, hindlimb suspension; MO‐C, Mineral oil supplemented group; MO‐HS, Mineral oil supplemented and hindlimb suspension group; EPA‐C, High eicosapentaenoic acid fish oil supplemented group; EPA‐HS, High eicosapentaenoic acid fish oil supplemented and hindlimb suspension group; DHA‐C, High docosahexaenoic acid fish oil supplemented group; DHA‐HS, High docosahexaenoic acid fish oil supplemented and hindlimb suspension group; P, pool containing a mixture with equal parts of all samples – used to normalize Ponceau S quantitative results; AU, arbitrary units.

The membranes were incubated with the primary antibodies for 12 h at 4°C and subsequently incubated for 1 h at 4°C with the secondary antibody conjugated to peroxidase (Cell Signaling Technology, Beverly, MA). After treatment with ECL detection reagent (Amersham/GE Healthcare, Waukesha, WI), the images were captured by the Amersham Imager 600 (Amersham/GE Healthcare) and quantified using the Image J software (NIH). Total loading of proteins for each sample, as indicated by the Ponceau S staining, was used to normalize the results (Romero‐Calvo et al. 2010; Gilda and Gomes 2013; Fortes et al. 2016) expressed in values relative to MO‐C. A pool sample, composed of equal parts of all experimental condition samples, was used for normalization among membranes (Fig. 1). The primary antibodies used were: p‐Akt at Ser 473 (9271), Akt (9272), p‐p70S6K at Thr 389 (9205), p70S6K (2708), p‐S6 at Ser 240/244 (5364), S6 (2217), p‐4EBP1 (eukaryotic translation initiation factor 4E‐binding protein 1) at Thr 37/46 (2855), 4EBP1 (9644), p‐GSK3‐beta (glycogen synthase kinase3‐beta) at Ser 9 (9323), GSK3‐beta (9315), p‐ERK (extracellular‐signal‐regulated kinases 1/2) at Thr 202/Tyr 204 (9101), and ERK (4695) from Cell Signaling Technology (Danvers, MA) and atrogin‐1 (AP2041) and MuRF1 (MP3401) from ECM Biosciences (Versailles, KY).

### Statistical analysis

Statistical analysis was performed using the GraphPad Prism^®^ software (version 4.01; El Camino Real, CA). Results are presented as mean ± standard error of the mean (SEM) and were analyzed by two‐way analysis of variance (ANOVA) followed by the Bonferroni post‐hoc test (for comparison between three or more groups). The differences between groups were considered significant for *P *<* *0.05. CSA of the soleus muscle fibers were not normally distributed, therefore, difference was considered significant when there was no overlap between 95% CI of the median (Gehrig et al. 2008; Pinheiro et al. 2012).

## Results

### Water ingestion, food intake, body weight, muscle wet weights, and fat depots

Daily water ingestion and daily food intake were not significantly different among the six groups (data not shown). The MO‐C group had a body weight gain of 156 ± 7.8 g, mean of 10 rats, over 4 weeks. HS reduced body weight gain in all groups (Table 2).

**Table 2:** Body weight and wet weight of skeletal muscles and fat depots

	Groups					
	MO‐C	MO‐HS	EPA‐C	EPA‐HS	DHA‐C	DHA‐HS
Body weight						
Initial body weight (g)	199 ± 4.5	203 ± 6.2	201 ± 9.9	200 ± 6.6	201 ± 5.7	207 ± 4.0
Increase of body mass after 4 weeks (g)	156 ± 7.8	92 ± 7.0^c^	136 ± 6.1	80 ± 5.4^c^	123 ± 7.7^y^	68 ± 4.2^c^, ^x^
Soleus muscle						
Wet weight (mg/mm tibia length)	3.6 ± 0.6	1.8 ± 0.09^c^	3.5 ± 0.1	1.7 ± 0.07^C^	3.3 ± 0.02	1.7 ± 0.10^c^
% Loss due to HS in wet weight		46	0	52		51
Gastrocnemius muscle						
Wet weight (mg/mm tibia length)	58 ± 1.3	42 ± 1.1^c^	55 ± 1.1	40 ± 1.1^c^	51.9 ± 1.02^y^	40.0 ± 0.13^c^
% Loss due to HS in wet weight		27		28		26
Tibialis anterior muscle						
Wet weight (mg/mm tibia length)	19 ± 0.5	17 ± 0.7^a^	18 ± 0.7	15 ± 0.6^c^	18 ± 0.6	15 ± 0.42^c^
% Loss due to HS in wet weight		13		18		20
EDL muscle						
Wet weight (mg/mm tibia length)	4.2 ± 0.1	3.5 ± 0.1^c^	4 ± 0.1	3.4 ± 0.1^b^	4 ± 0.1	3.3 ± 0.1^c^
% Loss due to HS in wet weight		16		16		20
Subcutaneous fat mass						
Wet weight (mg/cm LR)	188 ± 20.1	154 ± 13. 0	198 ± 17.4	138 ± 17.9	177 ± 9.1	139 ± 21.4
% Loss due to HS in wet weight		22		34		26
Epididymal fat mass						
Wet weight (mg/cm LR)	125 ± 4.2	142 ± 9.6	130 ± 14.7	109 ± 6.7	136 ± 5.7	93 ± 14.9^a^, ^y^
% Loss due to HS in wet weight		0.7		20		34
Retroperitoneal fat mass						
Wet weight(mg/cm LR)	91 ± 15.3	91 ± 11.6	112 ± 13.6	53 ± 8.8^b^, ^x^	95 ± 12.5	49 ± 11.2^a^, ^x^
% Loss due to HS in wet weight		4		55		51
Mesenteric fat mass						
Wet weight (mg/cm LR)	181 ± 14.7	161 ± 13.2	215 ± 22.6	139 ± 14.9^b^	181 ± 12.9	133 ± 12.2
% Loss due to HS in wet weight		15		39		31

Values are presented as mean ± SEM, *n* = 9–12 animals. The results were compared using two‐way ANOVA and Bonferroni post hoc test.

^a^ *P *<* *0.05; ^b^ *P *<* *0.01; ^c^ *P *<* *0.001 for: significant differences between hindlimb suspension groups and the respective controls. ^x^ *P *<* *0.05; ^y^ *P *<* *0.01 for: significant differences between the groups supplemented with High EPA or DHA fish oils versus that supplemented with mineral oil (MO‐C vs. EPA/DHA‐C or MO‐HS vs. EPA/DHA‐HS).

MO‐C, Mineral oil supplemented group; MO‐HS, Mineral oil supplemented and hindlimb suspension group; EPA‐C, High eicosapentaenoic acid fish oil supplemented group; EPA‐HS, High eicosapentaenoic acid fish oil supplemented and hindlimb suspension group; DHA‐C, High docosahexaenoic acid fish oil supplemented group; DHA‐HS, High docosahexaenoic acid fish oil supplemented and hindlimb suspension group; EDL, extensor digitorum longus; LR, length of the rat.

### Wet weights of the soleus, gastrocnemius, tibialis anterior, and EDL muscles, and of the subcutaneous, epididymal, retroperitoneal and mesenteric fat depots

HS reduced soleus muscle mass by approximately 50% in all groups compared with respective non‐HS‐treated animals (*P *˂ 0.001) (Table 2). The gastrocnemius muscle mass was also decreased by 26–27% (*P *˂ 0.001) in HS rats compared to non‐HS animals. Administration of high DHA fish oil (DHA‐C) reduced gastrocnemius muscle mass (normalized by tibia length) as compared to the MO‐C group (*P *˂ 0.01). HS reduced (*P *˂ 0.001) tibialis anterior muscle mass in all groups by 13–20% as compared to non‐HS animals. HS also reduced EDL muscle mass in all groups by 16–20% (Table 2). HS decreased the subcutaneous fat mass by 22% for MO, 34% for EPA, and 26% for DHA as compared to non‐HS animals. The epididymal fat mass was reduced in HS rats supplemented with either fish oil; 20% for EPA and 34% for DHA. HS did not alter the epididymal fat mass in MO animals. HS caused a decrease of the retroperitoneal fat mass in rats supplemented with either fish oil by 51–55% but it did not change in MO rats (Table 2).

### Composition of fatty acids in the gastrocnemius muscle

Omega‐6/omega‐3 fatty acid ratio was decreased in the gastrocnemius muscle from both fish oil supplemented groups when compared with the MO group (*P *<* *0.001). The values were: 4 ± 0.4 for MO‐C; 4 ± 0.5 for MO‐HS; 1 ± 0.1 for EPA‐C; 1 ± 0.1 for EPA‐HS; 1 ± 0.2 for DHA‐C and 1 ± 0.1 for DHA‐HS (data not shown).

### Glycogen, protein, and cytokine levels in soleus muscle

Contents of glycogen, total protein, and cytokines (TNF‐*α*, IL‐1*β*, IL‐6 and IL‐10) were measured using standard methods, but there was no significant difference among the experimental groups (data not shown).

### CSA of the soleus muscle and of soleus muscle fibers

**Figure 2:**
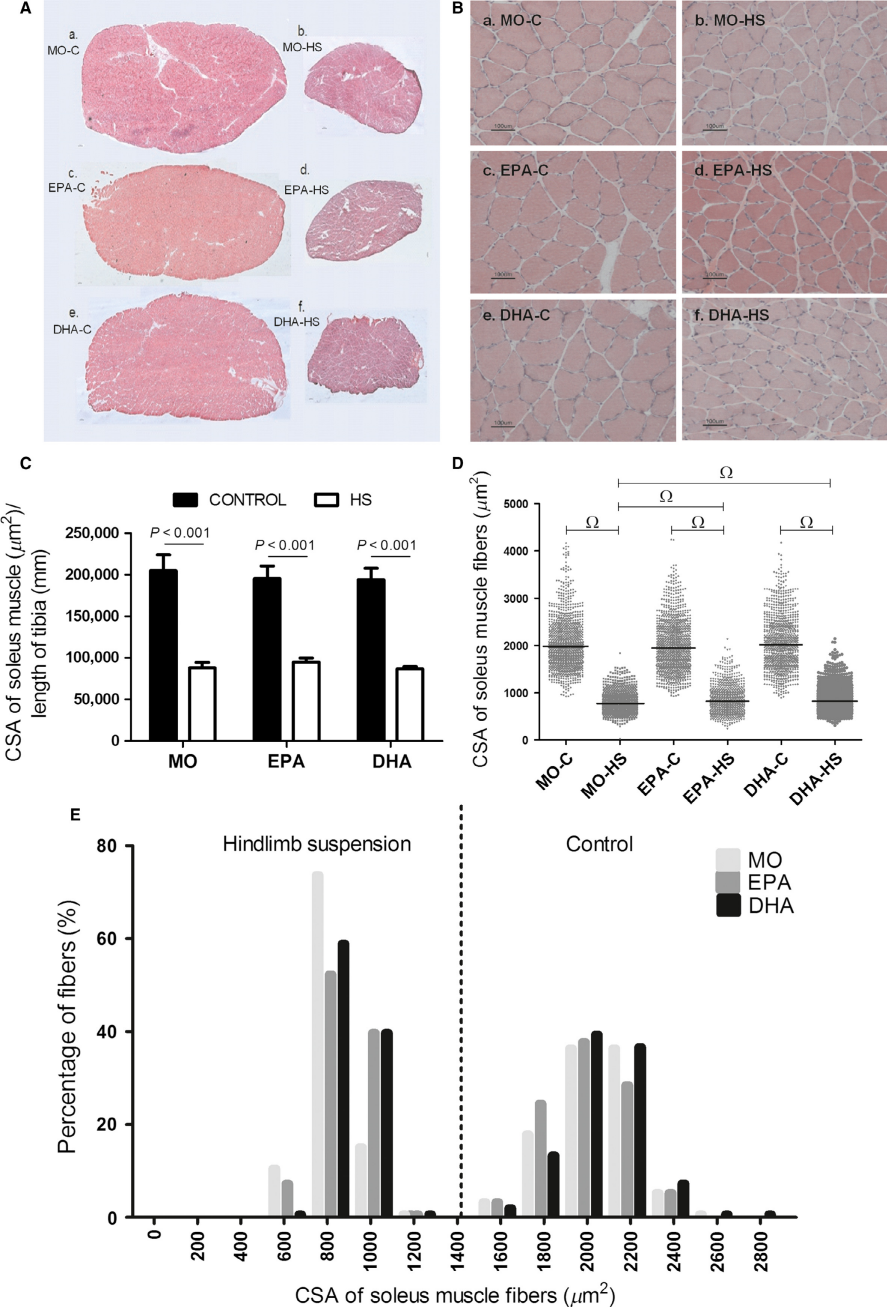
Cross‐sectional areas (CSA) of the soleus muscle and soleus muscle fibers. (A) Representative histological hematoxylin and eosin staining images of cross‐sectional areas of the soleus muscle. a. MO‐C; b. MO‐HS; c. EPA‐C; d. EPA‐HS; e. DHA‐C; f. DHA‐HS. Reference bar represents 100 *μ*m. (B) Representative histological hematoxylin and eosin staining images of cross‐sectional areas of soleus muscle fibers; a. MO‐C; b. MO‐HS; c. EPA‐C; d. EPA‐HS; e. DHA‐C; f. DHA‐HS. Reference bar represents 100 *μ*m. (C) Cross‐sectional area of the soleus muscle. Values are presented as mean ± SEM,* n* = 5–8 animals. The results were compared using two‐way ANOVA and Bonferroni post hoc test. (D) Cross‐sectional areas of soleus muscle fibers. Values are presented as scatter plot with median, *n* = 750–1199. The results were analyzed as previously described using the 95% CI of the median. (E) Distribution of the soleus muscle fibers according to the ranges of the areas of soleus muscle fibers: 0–2800 *μ*m^2^. MO‐C, Mineral oil supplemented group; MO‐HS, Mineral oil supplemented and hindlimb suspension group; EPA‐C, High eicosapentaenoic acid fish oil supplemented group; EPA‐HS, High eicosapentaenoic acid fish oil supplemented and hindlimb suspension group; DHA‐C, High docosahexaenoic acid fish oil supplemented group; DHA‐HS, High docosahexaenoic acid fish oil supplemented and hindlimb suspension group; MO, Mineral oil supplementation; EPA, High eicosapentaenoic acid fish oil supplementation; DHA, High docosahexaenoic acid fish oil supplementation; HS, hindlimb suspension. Ω, difference between groups.

CSA of the soleus muscle was markedly decreased (*P *<* *0.001) due to HS in all groups compared to non‐HS animals by 51–57% (Fig. 2A and C). HS also decreased CSA of the soleus muscle fibers in all groups by 57–61% (Fig. 2B and D). The treatment with either fish oil modified HS‐induced fiber CSA distribution feature. This effect, assessed quantitatively by the 95% CI of the median (Fig. 2D), was supported by qualitative analysis of the proportion of fibers in different ranges of CSA (Fig. 2E). Frequency distribution (Fig. 2E) was calculated and expressed as performed by others (Baehr et al. 2011; Ge et al. 2011; Kulakowski et al. 2011; Pistilli et al. 2011; Watson et al. 2012; Callahan et al. 2014). This is an alternative way to illustrate the trend of individual results depicted Figure 2D. Fish oil supplementation during HS increased the number of fibers in the range of 1000 *μ*m^2^ by approximately 140% (Fig. 2E). On the other hand, with respect to fibers in the range of 800 *μ*m^2^, supplementation with either fish oil during HS caused a reduction of approximately 25% when compared with MO animals.

### Activities of cathepsin L and 26S proteasome and content of carbonylated proteins in the soleus muscle

**Figure 3:**
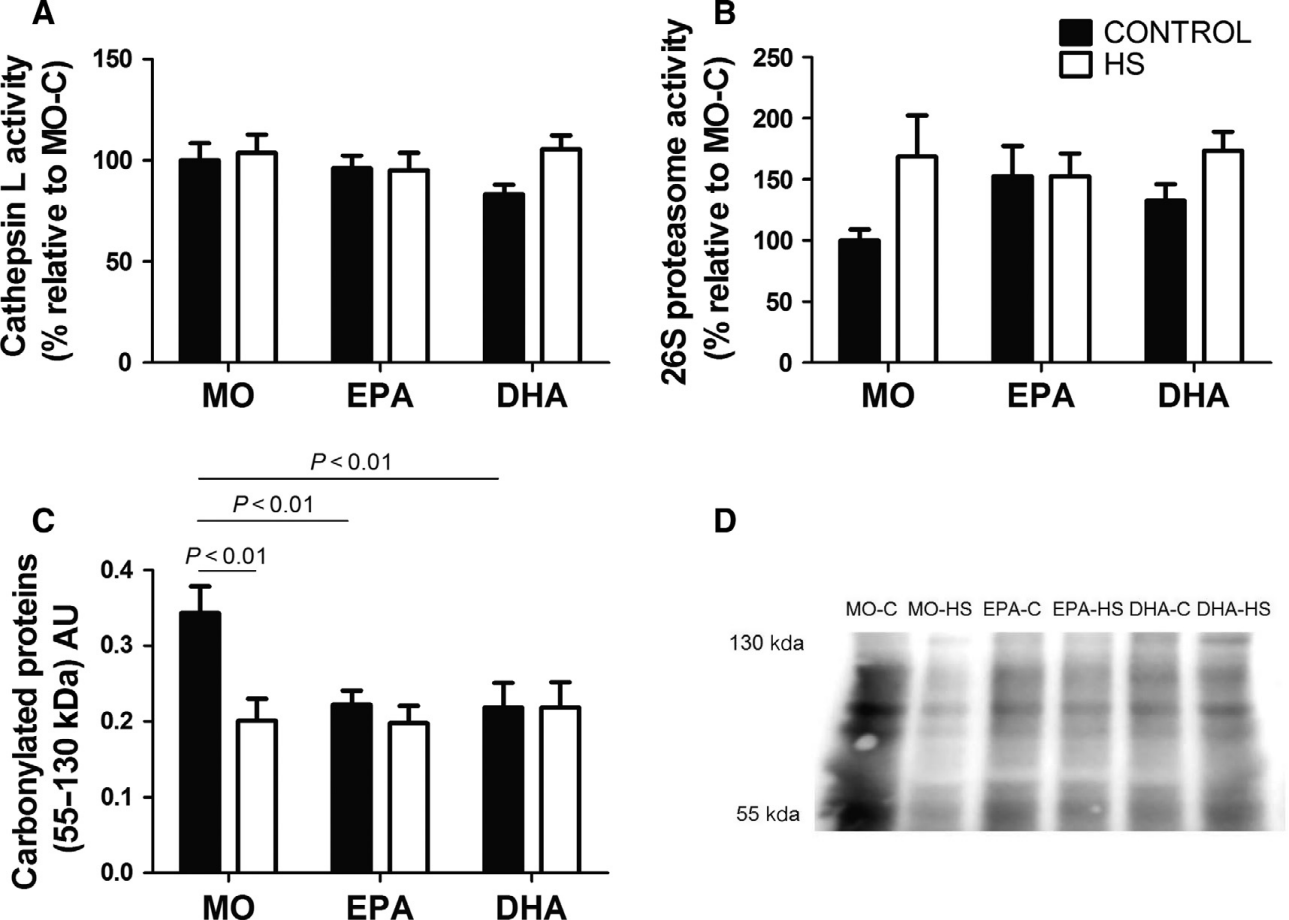
(A) The cathepsin L activity in soleus muscle (B) 26S proteasome activity in soleus muscle, (C) content of carbonylated proteins of soleus muscle and (D) representative image of the western blotting analysis of carbonylated proteins (55 KDa‐130 KDa). Values are presented as mean ± SEM on the basis of total protein loading as indicated by the Ponceau S measurement, *n* = 6–8 animals. The results were compared using two‐way ANOVA and Bonferroni post hoc test. MO, Mineral oil supplementation; EPA, High eicosapentaenoic acid fish oil supplementation; DHA, High docosahexaenoic acid fish oil supplementation; HS, hindlimb suspension; MO‐C, Mineral oil supplemented group; MO‐HS, Mineral oil supplemented and hindlimb suspension group; EPA‐C, High eicosapentaenoic acid fish oil supplemented group; EPA‐HS, High eicosapentaenoic acid fish oil supplemented and hindlimb suspension group; DHA‐C, High docosahexaenoic acid fish oil supplemented group; DHA‐HS, High docosahexaenoic acid fish oil supplemented and hindlimb suspension group; AU, arbitrary units.

The cathepsin L activity was not changed due to HS or supplementation with either fish oil in the soleus muscle (Fig. 3A). The activity of 26S proteasome was not significantly altered by the intragroup analysis (Bonferroni post hoc test). However, HS increased 26S proteasome activity (*P *˂ 0.05) compared with non‐HS animals; 69% for MO and 31% for DHA (Fig. 3B) as indicated by ANOVA (with no post hoc test difference). The content of carbonylated proteins in the soleus muscle was significantly changed by experimental conditions of the study as indicated by the Bonferroni post hoc test (MO‐C vs. MO‐HS; MO‐C vs. EPA‐C; MO‐C vs. DHA‐C, *P *˂ 0.01). EPA and DHA down‐regulated carbonylated proteins, but did not affect HS‐induced down‐regulation (Fig. 3C).

### Protein synthesis‐associated signaling in soleus muscle

**Figure 4:**
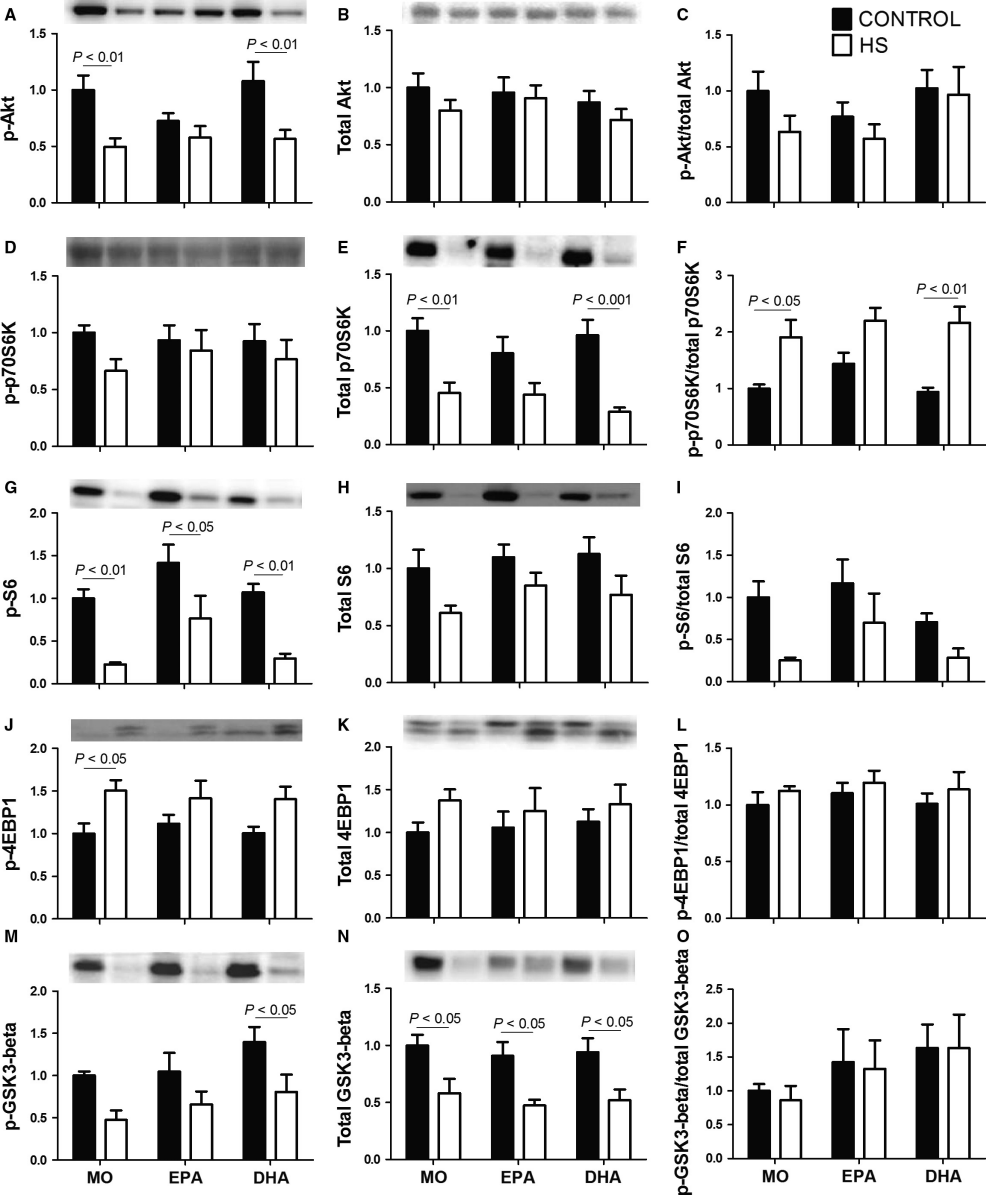
Contents of proteins associated with signaling pathway protein synthesis in the soleus muscle: (A) p‐Akt, (B) total Akt, (C) p‐Akt/Akt total ratio*,* (D) p‐p70S6k, (E) total p70S6k, (F) p‐p70S6k/p70S6k total ratio, (G) p‐S6, (H) total S6, (I) p‐S6/S6 total ratio, (J) p‐4EBP1, (K) total 4EBP1, (L) p‐4EBP1/4EBP1 total ratio, (M) p‐GSK3‐beta, (N) total GSK3‐beta, (O) p‐GSK3‐beta/total GSK3‐beta ratio. Values are presented as mean ± SEM on the basis of total protein loading as indicated by the Ponceau S measurements and expressed relative to MO‐C, *n* = 6–8 animals. The results were compared using two‐way ANOVA and Bonferroni post hoc test. MO, Mineral oil supplementation; EPA, High eicosapentaenoic acid fish oil supplementation; DHA, High docosahexaenoic acid fish oil supplementation; HS, hindlimb suspension; MO‐C, Mineral oil supplemented group; MO‐HS, Mineral oil supplemented and hindlimb suspension group; EPA‐C, High eicosapentaenoic acid fish oil supplemented group; EPA‐HS, High eicosapentaenoic acid fish oil supplemented and hindlimb suspension group; DHA‐C, High docosahexaenoic acid fish oil supplemented group; DHA‐HS, High docosahexaenoic acid fish oil supplemented and hindlimb suspension group.

The content of p‐Akt was reduced due to HS in the MO‐ and DHA‐rich fish oil groups (MO‐C vs. MO‐HS; DHA‐C vs. DHA‐HS, *P *˂ 0.01) (Fig. 4A). HS did not change the p‐p70S6K content although a slight reduction was found in the three groups: MO: 34%; EPA: 9.8%; DHA: 17% (Fig. 4D). The changes induced by HS in total p70S6K content (decrease) and p‐p70S6K/total p70S6K ratio (increase) were more pronounced in the MO and DHA groups (Fig. 4E and F). p‐S6 content was reduced in all groups due to HS; MO: 77%, EPA: 46%, and DHA, 72% (Fig. 4G). A decrease in total S6 and p‐S6/total S6 ratio (*P *˂ 0.05) due to HS (Fig. 4H and I) was indicated by ANOVA (with no post hoc test difference). The p‐4EBP1 content was significantly increased (*P *˂ 0.05) in the MO group due to HS but attenuated by either fish oil; MO: 50%, EPA: 27%, and DHA: 40% (Fig. 4J). A marked decrease of p‐GSK3‐beta in response to HS was observed; MO: 53%, DHA: 42%, and EPA: 37% (Fig. 4M). The total GSK3‐beta content was decreased due to HS in all groups by 42–48% (Fig. 4N).

### Protein degradation‐associated signaling in soleus muscle

**Figure 5:**
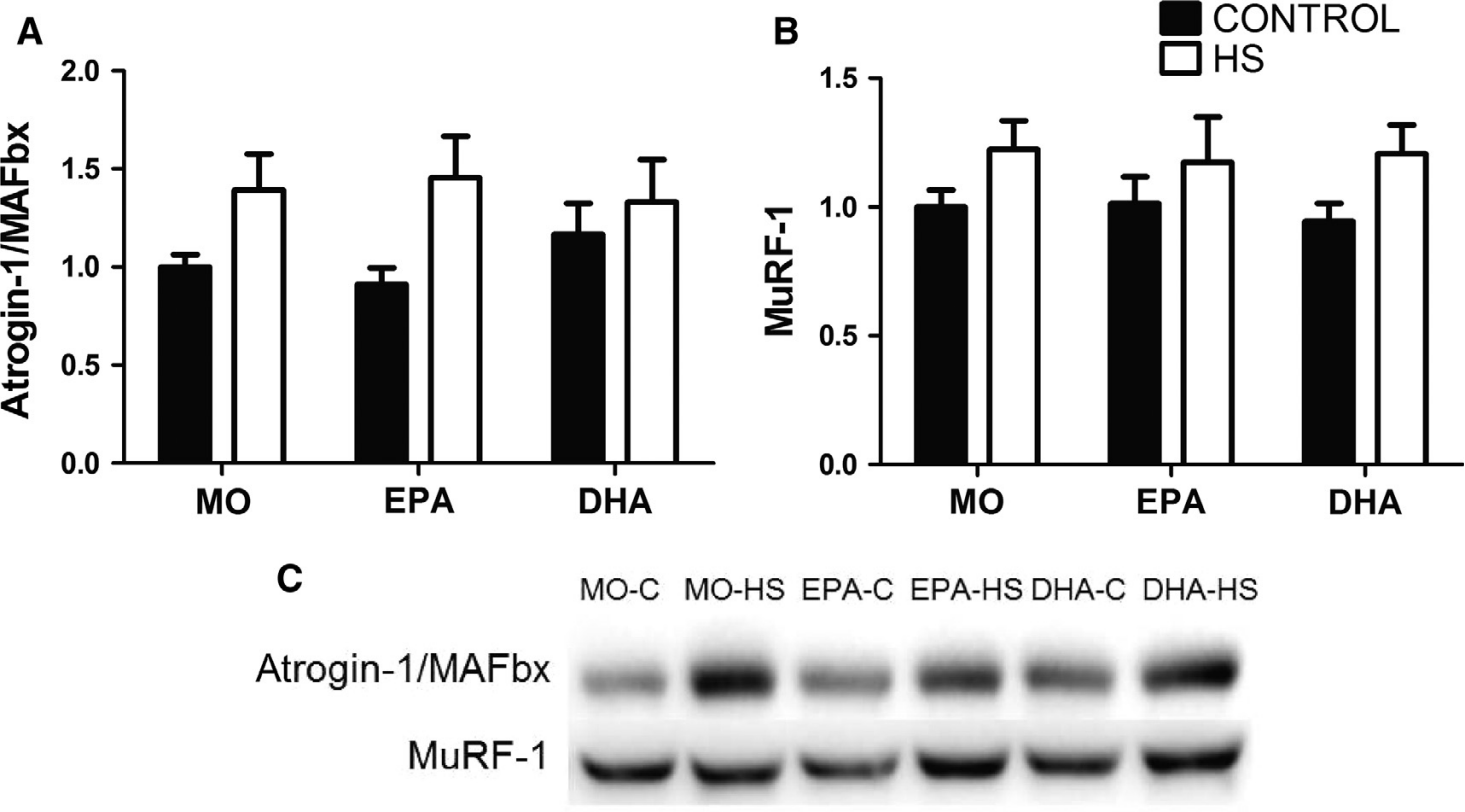
Contents of proteins associated with signaling pathway of protein degradation in the soleus muscle: (A) atrogin‐1/MAFbx, (B) MuRF‐1, (C) representative images of the western blotting analysis of atrogin‐1/MAFbx and MuRF‐1. Values are presented as mean ± SEM on the basis of total protein loading as indicated by the Ponceau S measurement and expressed relative to MO‐C, *n* = 7–8 animals. The results were compared using two‐way ANOVA and Bonferroni post hoc test. MO, Mineral oil supplementation; EPA,High eicosapentaenoic acid fish oil supplementation; DHA, High docosahexaenoic acid fish oil supplementation; HS, hindlimb suspension; MO‐C, Mineral oil supplemented group; MO‐HS, Mineral oil supplemented and hindlimb suspension group; EPA‐C, High eicosapentaenoic acid fish oil supplemented group; EPA‐HS, High eicosapentaenoic acid fish oil supplemented and hindlimb suspension group; DHA‐C, High docosahexaenoic acid fish oil supplemented group; DHA‐HS, High docosahexaenoic acid fish oil supplemented and hindlimb suspension group.

The content of atrogin‐1/MAFbx and MuRF‐1 was not significantly changed in the intragroup analysis (Bonferroni post‐hoc test). However, ANOVA (with no post hoc test difference) demonstrated that HS resulted in an increase in these proteins; MO: 39%, EPA: 60%, and DHA: 14% for atrogin‐1/MAFbx and MO: 22%, EPA: 17%, and DHA: 29% for MuRF‐1 (Fig. 5A and B).

### Changes in phosphorylated and total ERK 1 and 2 in soleus muscle

**Figure 6:**
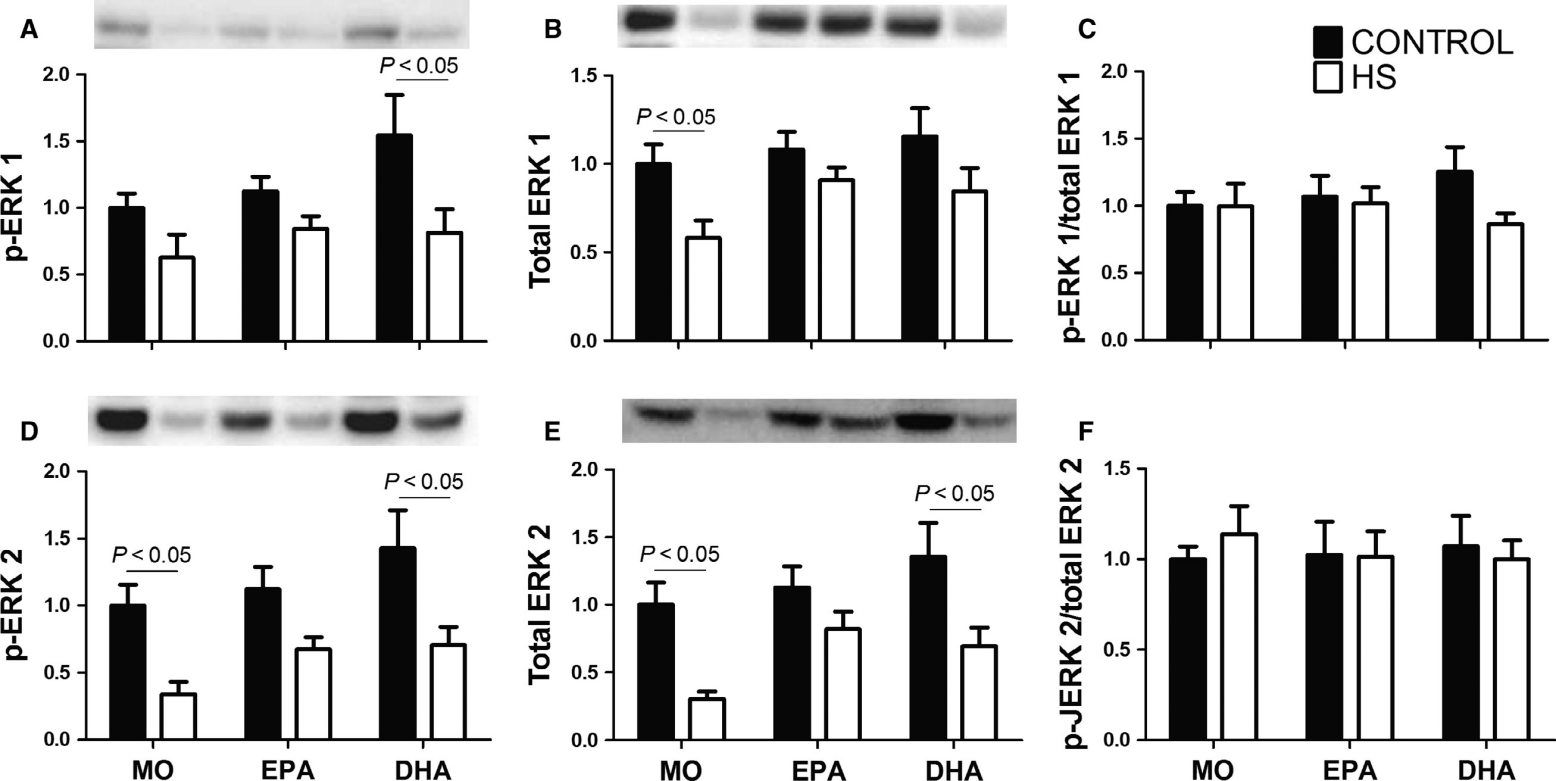
Contents of phosphorylated and total ERK 1 and 2 proteins in the soleus muscle: (A) p‐ERK 1, (B) total ERK 1, (C) p‐ERK 1/total ERK 1 ratio, (D) p‐ERK 2, (E) total ERK 2, (F) p‐ERK 2/total ERK 2 ratio. Values are presented as mean ± SEM on the basis of total protein loading as indicated by the Ponceau S measurements and expressed relative to MO‐C, *n* = 7–8 animals. The results were compared using two‐way ANOVA and Bonferroni post hoc test. MO, Mineral oil supplementation; EPA, High eicosapentaenoic acid fish oil supplementation; DHA, High docosahexaenoic acid fish oil supplementation; HS, hindlimb suspension; MO‐C, Mineral oil supplemented group; MO‐HS, Mineral oil supplemented and hindlimb suspension group; EPA‐C, High eicosapentaenoic acid fish oil supplemented group; EPA‐HS, High eicosapentaenoic acid fish oil supplemented and hindlimb suspension group; DHA‐C, High docosahexaenoic acid fish oil supplemented group; DHA‐HS, High docosahexaenoic acid fish oil supplemented and hindlimb suspension group.

HS reduced p‐ERK 1 content (*P *<* *0.05) in soleus muscle of the DHA‐treated group. The decrease in p‐ERK 1 content induced by HS was less pronounced following EPA fish oil administration (MO: 38%; EPA: 26%; DHA: 47%) (Fig. 6A). The total ERK 1 content was significantly decreased in the MO group due to HS (MO‐C vs. MO‐HS, *P *˂ 0.05). This latter effect was attenuated by treatments with either fish oil (MO: 42%; EPA: 16%; DHA: 27%) (Fig. 6B). HS decreased the contents of p‐ERK 2 and total ERK 2 proteins in the MO and DHA groups (*P *˂ 0.05). The decrease in p‐ERK 2 (MO: 69%; EPA: 39%; DHA: 50%) and total ERK 2 (MO: 69%; EPA: 27%; DHA: 31%) induced by HS was more pronounced in the MO group and only EPA treatment resulted in significant attenuation (Figs. 6D and E).

## Discussion

The level of skeletal muscle atrophy induced by HS varies with the type of muscle fiber affected and the length of treatment. Muscles predominantly made up of oxidative/slow twitch/type I fibers are associated with greater mass loss due to gravity withdrawn as in HS (Fitts et al. 2000; Bederman et al. 2013; Bodine 2013; Ciciliot et al. 2013). We have described greater effects of HS on soleus muscle that is composed mainly of oxidative/slow twitch/type I fibers (Soukup et al. 2002) and has anti‐gravitational roles (Bodine 2013). The HS effects were less pronounced in muscles with predominance of glycolytic/fast twitch/type II fibers. The level of muscle atrophy induced by HS was (greatest to weakest effect): soleus > gastrocnemius > tibialis anterior > EDL. These findings are in agreement with previous studies (Thomason & Booth 1990; Maki et al. 2012; Bodine 2013). Andrianjafiniony et al. (2010) reported a reduction of 41% in soleus muscle mass of Wistar rats induced by HS for 14 days, similar to this study (46%).

There was no marked effect of either fish oil on skeletal muscle mass preservation in this study, which may reflect the long duration of the HS protocol (high level of muscle atrophy), absence of inflammation, dose of fish oils used, and/or short period of supplementation. The beneficial effects of fish oil on skeletal muscle mass were reported in inflammation‐associated muscle wasting conditions as in cancer cachexia (Whitehouse et al. 2001; Tisdale 2007).

Oral supplementation of high EPA and high DHA fish oils markedly changed composition of fatty acids in gastrocnemius muscle. Decreased omega‐6 fatty acid and increased omega‐3 fatty acid levels that resulted in reduction of omega‐6/omega‐3 fatty acid ratio were also reported in previous studies (Hutchins‐Wiese et al. 2012).

HS caused a marked decrease in body weight as also observed by Hirose et al. (2008) in Wistar rats and Maki et al. (2012) in Sprague–Dawley rats. Animals submitted to HS initially have signs of stress because they are moved from a collective environment to an individual cage, which requires an adaptation period (Morey‐Holton and Globus 2002; Tsvirkun et al. 2012; Hanson et al. 2013). Control animals have then to be kept under identical cages and conditions (Morey‐Holton and Globus 2002). Herein, we followed the recommendations of the authors of previous studies (Thomason et al. 1987; Morey‐Holton and Globus 2002).

HS reduced fat mass as also previously reported by others (Hutchins‐Wiese et al. 2012; Lloyd et al. 2014). Hutchins‐Wiese et al. (2012) described HS for 2 weeks decreases epididymal fat mass in mice. They did not report changes in epididymal fat mass by the dietary conditions studied: control, moderate, and high omega‐3 fatty acid intake. Omega‐3 fatty acids have been reported to reduce fat mass in several animal experimental models (Belzung et al. 1993; Baillie et al. 1999; Pérez‐Matute et al. 2007; Buckley and Howe 2009) and humans (Parra et al. 2008; Buckley and Howe 2009; Bender et al. 2014; Moosheer et al. 2014). Herein, the decrease in the weight of epididymal, retroperitoneal, and mesenteric fat depots induced by HS was more pronounced following fish oil treatment as compared to mineral oil.

The CSA of the soleus muscle was decreased by more than 50% due to HS in all groups. Derbre et al. (2012) reported similar results in soleus muscle from rats submitted to HS for 14 days. HS also decreased CSA of the soleus muscle fibers and altered fiber CSA profile. Similar effect was reported by others (Andrianjafiniony et al. 2010; Maki et al. 2012; Liu et al. 2014). The results of muscle fiber CSA as described in Figure 2D (with indications of statistical significance) were also presented as frequency distribution in Figure 2E. Despite the limitations of the frequency distribution analysis, muscle disuse induced a marked decrease in muscle fiber CSA as expected. The treatments with both fish oils partially attenuated the trend to decrease muscle fiber area induced by HS. This statement is based on the increased proportion of muscle fibers with 1000 *μ*m^2^ and a decrease of muscle fibers with 800 *μ*m^2^ as a result of fish oil treatments in rats with HS. Although immunohistochemistry for type I and type II muscle fibers was not performed in this study, hindlimb suspension animal model has been reported to promote a decrease in the percentage of type I muscle fibers and an increase of type II muscle fibers (Caiozzo et al. 1997; Baldwin et al. 2013). So, the decrease in muscle fiber CSA induced by HS reported in this study parallels with the increase in type II muscle fibers described by others.

Cathepsin L activity, which indicates lysosomal proteolysis, is increased in skeletal muscle in disuse (Bechet et al. 2005). Taillandier et al. (1996) reported increased cathepsin L activity in rats submitted to HS for 9 days. Herein, no change in cathepsin L activity was observed probably due to the fact protein degradation intensity approaches control values after 14 days of HS (Hanson et al. 2013; Lloyd et al. 2014; Bodine et al. 2001). There are few studies on 20S and/or 26S portions of the ubiquitin proteasome system in HS (Bodine 2013). HS increased 26S proteasome and ubiquitin ligases, as atrogin‐1/MAFbx and MuRF1. The content of carbonylated proteins was reduced due to HS in MO rats. Treatments with fish oils, regardless HS, decreased the content of carbonylated proteins but did not promote further reduction when given to HS animals. An inverse relationship between proteasome activity and contents of carbonylated proteins has been reported (Cunha et al. 2012). Increased proteasome activity promotes degradation of misfolded proteins decreasing carbonylated protein levels. Derbre et al. (2012) did not report similar effects to those described in this study. The authors reported increased content of carbonylated protein in rat skeletal muscle after being suspended for 14 days. This difference may be due to the range of protein molecular weight investigated. Herein, proteins in the 55–130 KDa range were studied; similar used by others (Cunha et al. 2012).

HS decreases activity of signaling associated with protein synthesis and increases signaling associated with protein degradation (Bodine et al. 2001; Derbre et al. 2012; Maki et al. 2012; Bodine 2013; Liu et al. 2014; Lloyd et al. 2014). HS for 3 and 7 days progressively decreased Akt and S6 levels (Cannavino et al. 2014). On the other hand, at 7, 14, and 21 days of HS, there was a decrease in the activity of total protein synthesis and levels of S6 and 4EBP1 (Lloyd et al. 2014) in skeletal muscle. The decreased content of p‐Akt and total p70S6K induced by HS was partially reversed by treatment with high EPA fish oil. Fish oil promotes anabolism by enhancing insulin sensitivity and by activating the Akt‐mTOR‐S6 signaling pathway (Gingras et al. 2007). Akt also promotes GSK3‐beta phosphorylation that in turn attenuates the inhibition of eIF2B (eukaryotic initiation factor 2B) activity, so resulting in an increase of protein synthesis. We report herein a decrease of GSK3‐beta phosphorylation due to HS that was more pronounced in the high DHA fish oil group as compared to high EPA.

Changes in cell fatty acid composition impact on muscle cell proliferation and differentiation by modulating the activity of MAPKs (JNK 1/2, p38 and ERK 1/2) (Lee et al. 2009). Activation of JNK 1/2 and p38 and inhibition of ERK 1/2 have been postulated to occur during HS, which may be associated with induction of cell apoptosis during disuse‐induced muscle atrophy (Powers et al. 2007). Activation of p38 has been reported after two (Derbre et al. 2012) and three (Liu et al. 2014) weeks of HS. Increases of p‐ERK1 content and of p‐ERK2/total ERK2 ratio in mice soleus muscle after 3 weeks of HS have also been described (Liu et al. 2014). Supplementation with high EPA fish oil attenuated the decrease in the contents of phosphorylated and total ERK 1/2 induced by HS. However, little is known about ERK1/2 activation in skeletal muscle under such conditions (Powers et al. 2007). Contents of JNK 1/2 and p38 were not significantly different among the groups following treatment with either fish oil (data not shown).

You et al. (2010b) added cod liver oil to the diet of Sprague–Dawley rats during 2 weeks before inducing muscle atrophy for 10 days through leg immobilization (“local muscle atrophy”). The authors found an increase of atrogin‐1/MAFbx and MuRF1 contents and a decrease of p‐Akt/total Akt and of p‐p70S6K/total p70S6K ratios in MO rats, whereas fish oil‐enriched diet attenuated soleus muscle atrophy. The effect of reloading for 3 and 13 days after the period of leg immobilization was also investigated (2010a). The authors reported fish oil inhibits initial state of recovery after disuse by suppressing activation of the Akt‐p70S6K pathway and by reducing PGF2*α* content (2010a). Fappi et al. (2014) concluded that oral supplementation with fish oil (high EPA) in dexamethasone‐induced muscle atrophy has no positive effect. On the contrary, fish oil increased the expression of atrogenes (atrogin‐1/MAFbx and MuRF1) and reduced that of myogenin.

Differences were observed between the effects of the high EPA and high DHA fish oils in this study. High EPA fish oil decreased retroperitoneal fat depot and modulated the effects of HS on p‐Akt, total p70S6K, p‐p70S6K/total p70S6K ratio, p‐4EBP1, p‐GSK3‐beta, p‐ERK1, p‐ERK2, total ERK1, and total ERK2. High DHA fish oil per se attenuated body weight gain over 4 weeks and caused a reduction in gastrocnemius muscle mass, epididymal and retroperitoneal fat depots and attenuated changes induced by HS on p‐4EBP1 and total ERK1 levels. Therefore, the effects of EPA‐rich fish oil on signaling pathways associated with protein synthesis were clearly more pronounced than those by DHA‐rich fish oil. Both EPA‐ and DHA‐rich fish oils did not exhibit marked effect on skeletal muscle mass loss induced by HS under the conditions of this study.

## Conflict of Interest

Marzuca‐Nassr GN, Vitzel KF, de Sousa LG, Murata GM, Crisma AR, Rodrigues CF Jr, Abreu P, Torres RP, Mancini‐Filho J, Hirabara SM, Newsholme P, and Curi R have no conflict of interest with respect to this study.
